# 12-*oxo*-Phytodienoic Acid: A Fuse and/or Switch of Plant Growth and Defense Responses?

**DOI:** 10.3389/fpls.2021.724079

**Published:** 2021-08-17

**Authors:** Wenshan Liu, Sang-Wook Park

**Affiliations:** Department of Entomology and Plant Pathology, Auburn University, Auburn, AL, United States

**Keywords:** cyclophilin 20-3, fitness tradeoffs/balances, light-dependent redox reactions, redox signaling, retrograde signaling

## Abstract

12-oxo-Phytodienoic acid (OPDA) is a primary precursor of (-)-jasmonic acid (JA), able to trigger autonomous signaling pathways that regulate a unique subset of jasmonate-responsive genes, activating and fine-tuning defense responses, as well as growth processes in plants. Recently, a number of studies have illuminated the physiol-molecular activities of OPDA signaling in plants, which interconnect the regulatory loop of photosynthesis, cellular redox homeostasis, and transcriptional regulatory networks, together shedding new light on (*i*) the underlying modes of cellular interfaces between growth and defense responses (e.g., fitness trade-offs or balances) and (*ii*) vital information in genetic engineering or molecular breeding approaches to upgrade own survival capacities of plants. However, our current knowledge regarding its mode of actions is still far from complete. This review will briefly revisit recent progresses on the roles and mechanisms of OPDA and information gaps within, which help in understanding the phenotypic and environmental plasticity of plants.

## Phyto-Oxylipins: Jasmonate Signaling in Plants

Oxylipins, the oxygenated derivative of fatty acids (FAs), are critical signal molecules in diverse physiological processes in life, including plants and animals (Marnett, 2008). In plants, oxylipins are involved in a layer of defense and ontogenetic pathways, while mammalian oxylipins (eicosanoids) control intricate regulatory mechanisms in immunity, functioning as messengers in the central nervous system, and participating in the resolution process following tissue injury (Funk, 2001; Mosblech et al., 2010). Recent studies, moreover, have illuminated the medicinal values of phyto-oxylipins, presenting their anticancer, anti-inflammatory, and antioxidative activities (Flescher, 2007; Dang et al., 2008; Taki-Nakano et al., 2014). Noticeably, the molecular components and metabolic pathways, involved in oxylipin biogenesis and signaling, share common ancestry and evolutionary processes across Kingdoms (Marnett, 2008). Hence, uncovering the modes of actions associated with oxylipins will not only assist the development of agricultural strategies in advancing disease resistance and stress adaptation, as well as yield and biomass increases in plants, but also assist the improvement of drug development through facilitating the rational design of more potent and safe anticancer (and anti-inflammation) drugs. However, our current knowledge regarding oxylipin signaling is still incomplete, despite decades of investigations (Funk, 2001; Mosblech et al., 2010).

Lately, molecular underpinnings have been investigated for 12-oxo-phytodienoic acid (OPDA) signaling in plant defense responses. OPDA is a primary precursor of the jasmonate family of oxylipins, which includes jasmonic acid (JA) and its precursors and derivatives. Jasmonates are derived from trienoic-FA *via* the octadecanoid pathway in the chloroplasts. Lipase-mediated oxidation of trienoic-FA leads to the release of OPDA that travels to the peroxisomes through plastid envelope proteins (e.g., OPDAT1 and JASSY, Guan et al., 2019; Zhao et al., 2020) and/or peroxisomal ATP-binding transporters (e.g., COMATOSE, Theodoulou et al., 2005) and undergoes β-oxidations to form JA. JA can be further metabolized to several derivatives, including JA-isoleucine (JA-Ile), JA-tryptophan (JA-Trp), methyl-JA, and hydroxyl-JA. Signaling of these jasmonate molecules then controls a large number of gene expressions in the nucleus and mediates defense (adaptive) responses to various forms of biotic and abiotic stresses, including microbial pathogens and insect herbivores, tissue injury, and light damage. Jasmonate signaling also plays essential roles in reproduction and other developmental processes such as senescence, root growth and tuberization, fruit ripening, and tendril coiling (reviewed in Acosta and Farmer, 2010; Pieterse et al., 2012). These important, yet diverse activities of jasmonates must be tied to their versatility as major molecular and cellular modulators.

The most well-characterized jasmonate-associated signaling pathway revolves around JA-Ile. Once it is produced, JA-Ile binds a F-box protein, CORONATINE INSENSITIVE 1 (COI1, a part of SCF ubiquitin E3 ligase). This complex then binds and ubiquitinates jasmonate ZIM-domain (JAZ) proteins, which are negative transcription regulators of JA-responsive genes (JRGs). Thus, JAZ degradation by 26S proteasomes frees transcription factors (TFs, e.g., bHLH-containing MYCs) and allows subsequent gene expressions (Chini et al., 2007; Thines et al., 2007). Jasmonate signaling, however, must involve a much more complex network, given that a number of JRGs respond independently of COI1 (Devoto et al., 2005). For example, JA induction of *GRX480* and *AOC3* is mediated *via* a COI1-independent MYC2 regulatory pathway, whereas JA-activated MAP kinase cascades (e.g., *MPK1, MPK2* and *BIK1*) and *GST25* are regulated in a COI1- and/or MYC2-independent manner (Veronese et al., 2006; Ortiz-Masia et al., 2007; Stotz et al., 2013). In addition, OPDA is capable of triggering autonomous signaling pathways that regulate unique subsets of JRGs, coordinated with and without the canonical JA pathway (Taki et al., 2005). OPDA signaling is presumed to be independent of COI1, as it is unable to bind the COI1/JAZ complex (Thines et al., 2007). However, OPDA induction of *PHO1;H10* needs COI1 activity (Ribot et al., 2008), suggesting additional layers of complexity in jasmonate signaling. In fact, ancestral plants such as the bryophyte *Marchantia polymorpha* are able to synthesize only a set of OPDAs (OPDA, dinor-*cis*-OPDA and dinor-*iso*-OPDA), but not JA/JA-Ile, though their genomes still express a functional COI1 (Monte et al., 2018). Hence, *M. polymorpha* deploys OPDAs, instead of JA-Ile, to activate COI1/JAZ signaling for defense activations and growth processes (Monte et al., 2018, 2019). The other study also established a distinct role of JA-Trp conjugate, linking jasmonate with auxin signaling (Staswick, 2009), further supporting the notion that distinct messages sent out by specific jasmonate coordinate essential molecular and cellular processes.

## Biosynthesis of OPDA and its Derivatives

As alluded, jasmonates are synthesized in the chloroplasts from oxygenized FAs, linolenic acid (18:3) and hexadecatrienoic acid (16:3), that are stored mostly as the esterified monogalactosyldiacylglycerol (MGDG). The first step, hydroperoxidation, is began by 13-lipoxygenases adding molecular oxygen to 18:3 and 16:3 and forming 13(*S*)-hydroperoxy-octadecatrienoic acid and 11(*S*)-hydroperoxy-hexadecatrienoic acid, respectively. These compounds are then transformed *via* allene oxide synthase into (*13S*)12,13-epoxy-octadecatrienoic acid and (11*S*)10,11-epoxy-octadecatrienoic acid, which are subsequently cyclized through allene oxide cyclase to yield *cis*-(+)-OPDA and dinor-OPDA (collectively, OPDA); containing a reactive electrophilic α,β-unsaturated carbonyl group. These metabolic pathways are known to be activated in response to various herbivories and microbial pathogens, as well as abiotic stresses such as extreme temperatures and tissue injury (Stintzi et al., 2001; Kourtchenko et al., 2007; Vu et al., 2012; Bosch et al., 2014a,b; Monte et al., 2020). Some portion of OPDA is then further derivatized to a glutathione (GSH) conjugate, or galactolipids (later named “arabidopsides”) by its binding with MGDG and digalactosyl DG (Hisamatsu et al., 2003, 2005; Davoine et al., 2005, 2006; Andersson et al., 2006; Buseman et al., 2006). The biological roles of OPDA-GSH and arabidopsides are yet largely elusive, but have been hypothesized as the vacuolar delivery and storage forms, respectively, in maintaining the cellular-level homeostasis of OPDA to avoid their potential toxicity and/or negative effects on physiol-molecular processes in plants (Böttcher and Pollmann, 2009; Ohkama-Ohtsu et al., 2011). Alternatively, recent studies have been suggested that arabidopsides could interact with plant plasma membrane lipids such as glycosyl inositol phosphor ceramides, which thus lead them to locate and modify membrane organizations, and such changes could signal defense mechanism activations (Genva et al., 2019).

## Signaling of OPDA in Plant Defense Responses

**Figure 1:**
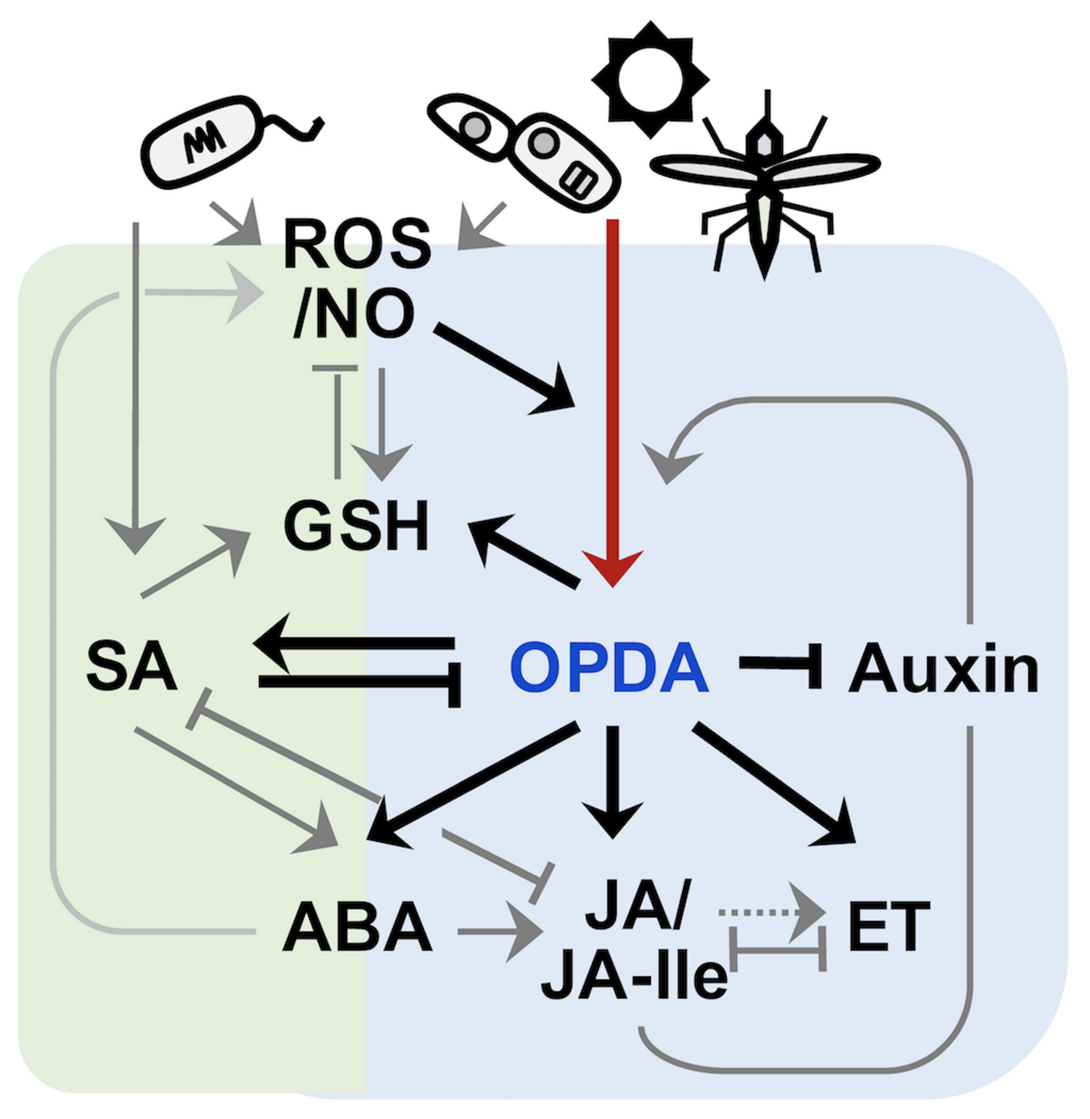
Schematic crosstalk of OPDA with the signaling pathways of other phytohormones. OPDA and JA/JA-Ile (collectively, jasmonate) signaling activates defense responses to various forms of biotic and abiotic stresses, including necrotroph infection, insect attack, wounding, and UV damage, whereas salicylic acid (SA) signaling confers disease resistance against mostly biotrophic pathogens. Once recognized “non-self,” plants rapidly produce ROS including nitric oxide (NO) (Huang et al., 2004; Wang et al., 2013), which in turn actuates jamonate biosynthesis (Palmieri et al., 2008). OPDA signaling then stimulates and coordinates with different defense signaling, e.g., JA/JA-Ile, SA, ABA, ethylene (ET), and GSH pathways (Taki et al., 2005; Park et al., 2013; Dave et al., 2016; Varsani et al., 2019; Scalschi et al., 2020), while suppressing growth hormone (e.g., auxin) signaling (Taki et al., 2005) to optimize defense capability of plants. On the other hand, JA/JA-Ile signaling can feedback induce own jasmonate biosynthesis (Taki et al., 2005), whereas SA signaling antagonizes both OPDA and JA/JA-Ile signaling (Leon-Reyes et al., 2010; Shim et al., 2013; Wei et al., 2014).

In plants, OPDA signaling plays intrinsic roles in activating and fine-tuning defense (adaptive) responses against an array of biotic and abiotic stresses, as well as growth processes (Böttcher and Pollmann, 2009; Dave and Graham, 2012; Maynard et al., 2018). Its distinctive activity in plant defense activations was first described by the pathoanalyses of a mutant Arabidopsis plant (*opr3*) arresting the conversion of OPDA to JA/JA-Ile (Stintzi et al., 2001). WT-like resistance of *opr3*, in contrast to enhanced susceptibility in other mutants disrupting trienoic-FA biosynthesis (*fad3*/*7*/*8*) and the octadecanoid pathway (*dde2* and *aos*), against fungal pathogens (*Alternaria brassicicola* and *Scerotinia sclerotiarum*) and an insect herbivory (*Bradysia impatiens*), underlined a critical activity of OPDA signaling in plant disease resistance in the absence of JA/JA-Ile (Stintzi et al., 2001; Zhang and Turner, 2008; Stotz et al., 2011). Following studies with genetically modified (GM) plants reducing or impairing JA productions (*OPR3-RNAi, SiOPR3*s, and *opr7opr8*) or enhancing OPDA accumulations (*OPR3ox*) further substantiate that OPDA signaling is essential for the full activation of basal defense responses in tomato, maize, and rice against microbial and/or pest attacks such as *Botrytis cinerea*, tobacco hornworm (*Manduca sexta* larvae), beet armyworm (*Spodoptera exigua* larvae), brown plant hopper (*Nilaparvata lugens*), green peach aphid (*Myxus persicae*), and corn leaf aphid (*Rhopalosiphum maidis*) (Bosch et al., 2014a,b; Guo et al., 2014; Scalschi et al., 2015; Varsani et al., 2019; Grover et al., 2020b; Wang et al., 2020). Upon their infections, OPDA is induced rapidly in the chloroplasts and triggers the retrograde signaling toward the nucleus, which coordinates large-scale changes in defense gene expressions (Taki et al., 2005). These then lead to (*i*) the spatiotemporal induction of protease inhibitors (PIs) such as miraculin-like proteins, which likely serve as antinutrients against insect attackers by reducing their digestibility of dietary proteins (Felton, 2005; López-Galiano et al., 2017), (*ii*) the actuation of other hormone and metabolite biosynthesis (Figure 1) in maximizing defense capacity and survival of plants, and (*iii*) the stimulation of callose deposition (Scalschi et al., 2015; Varsani et al., 2019), a multifaceted cell wall barrier developed at the sites of infection, preventing the cell-to-cell spread of microbes and limiting the feeding capacity and colonization of insects (Luna et al., 2010; De Storme and Geelen, 2014). OPDA signaling appeared to trigger abscisic acid (ABA) accumulations (Dave et al., 2016) that activate a NADPH oxidase subunit of RBOHF (Respiratory Burst Oxidase Homolog Protein F) leading to transient reactive oxygen species (ROS) productions (Sirichandra et al., 2009; Figure 1) and in consequence stimulating callose synthesis (Luna et al., 2010). However, OPDA signal alone did not elevate the expression levels of callose synthase gene such as *Tie-dyed2* in maize, suggesting rather the need of additional or alternative, yet unknown, defense and/or OPDA-inducible element(s), perhaps free thiols such as GSH and glucosinolates (Park et al., 2013; Zhou and Memelink, 2016; Varsani et al., 2019).

Indeed, it is still largely elusive how OPDA is perceived for signaling. Recently, in search of OPDA derivatives potentially binding SCF^COI^, OPDA conjugated with Ile (OPDA-Ile) was identified in Arabidopsis (Floková et al., 2016) and described its ability to induce OPDA-responsive genes (ORGs) such as *GRX480* and *ZAT10* in JA/JA-Ile-deficient mutant (*opr3* and *jar1*) plants (Arnold et al., 2016). The latter suggests that OPDA-Ile is a bioactive signal and conveys JA/JA-Ile-independent, OPDA-dependent signaling pathway. OPDA-Ile is though only active under specific conditions as it was found exclusively in wounded leaves of flowering plants (Floková et al., 2016). It would be interesting to delineate mechanisms underlying the perception of OPDA-Ile and its cross-networking with other OPDA signaling pathways (see “Summary: Mode of Action of OPDA Signaling”).

Besides its roles in local defenses, a new study has proposed that OPDA is a long-distance signal for “induced systemic resistance” (ISR) (Wang et al., 2020), a state of heightened defense that is activated throughout the plant following an initial encounter with plant growth-promoting rhizobacteria/fungi (PGPR/F) (Pieterse et al., 2014). Their oxylipin profiling of xylem saps collected from ISR-induced maize leaves detected uniquely OPDA and KODA (α-ketol-octadecadienoic acid). In addition, the transfusion of OPDA and KODA into naïve plants led to develop systemic resistance against an anamorphic fungus *Collectotrichum graminicola* in a dose-dependent manner (Wang et al., 2020), together proposing their role in conveying ISR signaling. A caveat is that OPDA appears to be stationary exhibiting little or no distal accumulation under pathogen attacks (Christensen et al., 2015). This speculates if an ISR receptor of OPDA is present in xylem. Alternatively, OPDA may be rapidly converted to and activate JA/JA-Ile signaling, upon arrival to systemic tissues, for priming systemic defense (Koo et al., 2009; Bosch et al., 2014a). In this context, an earlier grafting experiment using WT and JA/JA-deficient (*OPR3-RNAi*) plants showed that OPDA can substitute for JA/JA-Ile in the local induction of defense gene expressions, but the production of JA/JA-Ile is required for systemic responses (Bosch et al., 2014b). It will be intriguing to find out whether OPDA is “truly” moved from local to vascular to systemic tissues, and if OPDA can autonomously signal ISR priming or is converted to JA/JA-Ile for ISR development. In addition, we cannot still rule out a potential role of phloem in channeling a mobile signal of ISR development (Varsani et al., 2019). Perhaps, ISR may also involve multiple signals and transduction pathways as does in systemic acquired resistance (Klessig et al., 2018).

On the other hand, a recent report argued that only a biologically active jasmonate molecule is JA-Ile (Chini et al., 2018). Using a new mutant allele (*opr3-3*) completely lacking OPR3 reductase activity, the study demonstrated the increased accumulation levels (~fifteen-folds) of non-reduced cyclopente none, 4,5-didehydrojasmoante, in *opr3* and its provisional reduction to JA by one of OPR3 isoforms, OPR2 reductase, together postulating that WT-like resistance of *opr3* is actuated not by OPDA signaling, rather by COI1-dependent JA-Ile signaling. However, the OPR3-independent pathway of JA biosynthesis appeared to contribute to the accumulation of dismal amounts of JA-Ile (<2.0 % [less than its basal levels] of WT) under stress conditions, while conferring tenable strength defense responses against pathogen infections, prompting speculation that *opr3-3* mutants may exert alternative, OPDA-associated defense pathways. In fact, *coi1* mutants feedback suppress JA biosynthesis so that lack stress-induced OPDA and JA accumulations (Chung et al., 2008; Park et al., 2013). Thus, *coi1*-like increased susceptibility shown in *coi1*/*opr3-3* against insect and fungal attacks (Chini et al., 2018) might be, not because WT-like resistance of *opr3-3* requires COI1, due to auxiliary side effects led by double mutagenesis, perhaps lowering the level threshold of OPDA and JA-Ile signaling.

## Signaling of OPDA in Plant Growth and Developmental Processes

An earlier study of COMATOSE, a peroxisomal ATP-binding cassette transporter, and its mutant plants (*cts*) disrupted the transport of OPDA into the peroxisome, where JA biosynthesis occurs, illuminated a critical activity of OPDA signaling in coordinating seed germination and dormancy (Russell et al., 2000). The mutant *cts* seeds exhibited increased accumulation level of OPDA and low germination rates (Russell et al., 2000; Dave et al., 2011), while exogenous OPDA applications stimulated the repression of the germination of WT seeds (Dave et al., 2011). Such an inhibitory effect of OPDA signaling is perhaps mediated through its activation of ABA biosynthesis by upregulating the expression of an ABA biosynthesis gene (*ABA1* and *ABA-deficient 1*) and an inducer (*RGL2, Repressor of Gibberellic Acid-like 2*) of RING-H2 XERICO (ABA biosynthesis regulator) (Ko et al., 2006; Piskurewicz et al., 2008; Dave et al., 2016). OPDA and ABA both are then able to induce and/or stabilize the activity of GRL2 and ABI5 (ABA insensitive 5) bZIP TF, which in subsequence promotes the expression of *MET* (*Mother-of FT and TFL1*), an inhibitor of seed germination or early seedling growth (Skubacz et al., 2016; Vaistij et al., 2018), so that it suppresses seed germinations (Dave et al., 2016; Barros-Galvão et al., 2019). Two hormones, however, displayed different mechanistic outcomes that ABA signal ruptures seed coats and endosperm tissues, whereas OPDA-treated seeds keep intact endosperm and seed coats (Dave et al., 2011), indicating that OPDA signal, besides coordinating ABA biosynthesis/signaling, can execute its autonomous, unique regulatory metabolic pathways in plant organismal development.

**Figure 2:**
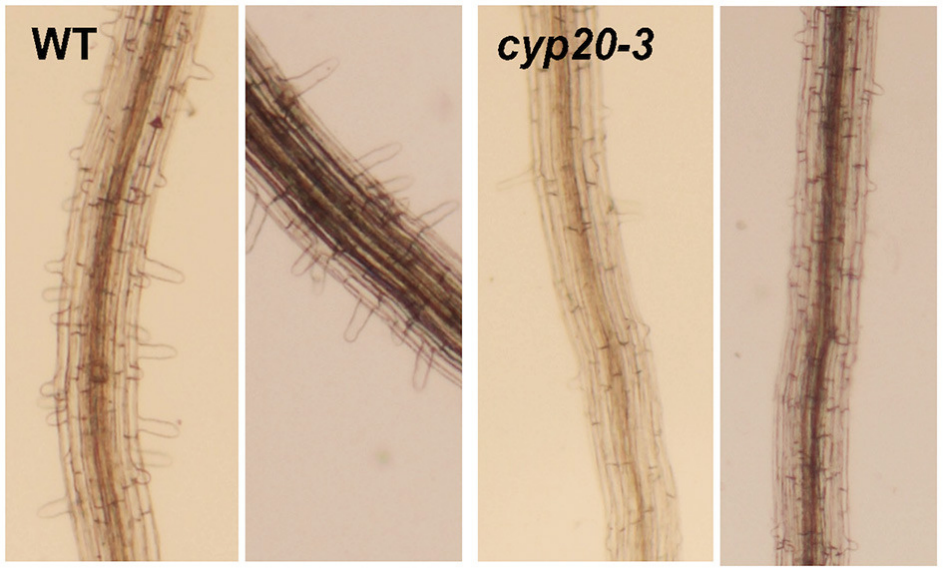
Important roles of OPDA signaling in root morphogenesis. OPDA signaling mutant Arabidopsis (*cyp20-3*) demonstrated the impairment of root hair growth.

OPDA-responsive ABA accumulations also convey the inhibition of root growth and morphogenesis in plants (Mueller et al., 2008; Park et al., 2013; Sun et al., 2018; Vissenberg et al., 2020). ABA could suppress primary root growth and lateral root branching, mediated *via* balancing the cellular homeostasis of several growth components (Arc et al., 2013; Sun et al., 2018) that enhance the production of ROS, Ca^2+^, and ethylene but reduce auxin levels (Wang et al., 2002; He et al., 2012; Jiao et al., 2013; Luo et al., 2014). These changes then stimulate the expression of *PLETHORA* TFs, rhizotatic regulators, and some cell cycle-related genes (e.g., *Cyclin-dependent Protein Kinase* and *Cell Cycle B-type Cyclins*), thus affecting DNA replication, cell division, and cell elongation in roots and inhibiting root growth (Wang et al., 2008, 2011; Yin et al., 2009; Xu et al., 2010; Hofhuis et al., 2013; Yao et al., 2013). However, the effects of OPDA signal on roots did also not entirely depend on ABA signaling. Our recent study indicated that OPDA signaling could act as a positive regulator in root growth and development (Figure 2). Disruption of OPDA signaling in Arabidopsis (*cyp20-3*, Park et al., 2013) engendered the impairment of root hair growth. It is though unclear if the opposite is correct; the increased accumulation level of OPDA under stress conditions could enhance root growth and branching; further studies are needed to reconstitute the complete, functional networks of OPDA signaling in plant growth and development.

## A Mode of OPDA Signaling by its Binding Protein Cyclophilin 20-3

**Figure 3:**
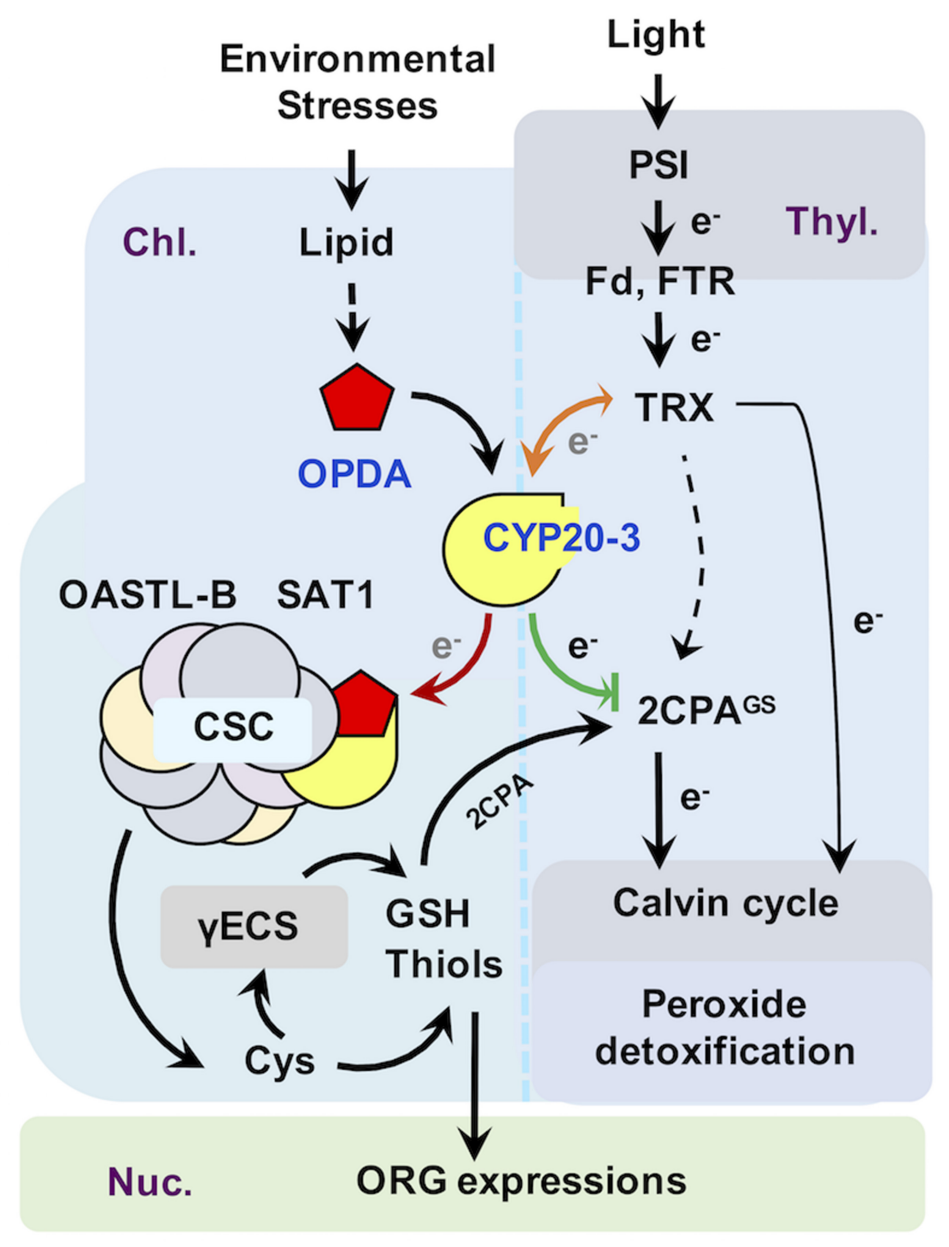
Proposed model of CYP20-3 as a regulatory hub in the interplay between light and OPDA signaling. In optimal conditions, CYP20-3 relays light signaling in buffering cellular redox homeostasis, whereas, in stressed states, CYP20-3 interfaces light and OPDA signaling, which fine-tunes plant fitness between growth (light-dependent detoxification and Calvin cycle) and defense response (redox-mediated retrograde signaling). Colored arrows indicate the enhanced interactions of CYP20-3 with TRXs (orange), SAT1 (red), and 2CPA (green) during stress (OPDA-signaling) defense responses. Hypothesized passage of electron (e^−^) transfers is noted in gray.

Previously, our search of jasmonate receptors uncovered that a small plastid protein, cyclophilin 20-3 (CYP20-3), can physically interact with OPDA, and its T-DNA insertion mutant Arabidopsis (*cyp20-3*) attenuates the expression of ORGs (Park et al., 2013). The Arabidopsis genome encodes 29 CYP and CYP-like proteins, belonging to the family of, namely, immunophilins that possess binding abilities toward immunosuppressive drugs, cyclosporin A, and functions in broad ranges of cellular processes, including transcriptional regulation, organogenesis, photosynthetic and hormone signaling pathways, stress adaptation, and defense responses (Dos Santos and Park, 2019). CYP20-3 is the only isoform localized in the chloroplast stroma and acts as a dual-enzyme able to chaperone protein folding (peptidyl-prolyl *cis*-*trans* isomerase; PPlase) and transfers electrons (e^−^) to peroxide substrates (reductase) in cysteine (Cys) biosynthesis (i.e., sulfur assimilation; Romano et al., 2004; Laxa et al., 2007; Dominguez-Solis et al., 2008; Park et al., 2013). In line with this scenario, OPDA, once accumulated under stress states, binds and stimulates CYP20-3 to form a complex with serine acetyltransferase1 (SAT1), which triggers the formation of a hetero-oligomeric Cys synthase complex (CSC) with *O*-acetylserine(thiol)lyase B (OASTL-B) (Figure 3, left side). CSC formation then leads to the production of Cys and subsequently thiol metabolites (e.g., GSH), which builds up cellular reduction potentials. The enhanced redox capacity in turn coordinates the expression of a subset of ORGs that activate and calibrate pathogen defense and stress adaptation processes. Thus, the KO of *CYP20-3* (*cyp20-3*) displays enhanced susceptibility against necrotrophic fungal (e.g., *A. brassicicola* and *B. cinerea*) and oomycete (*Pythium irregulare*) infections, as well as nematode (*Meloidogyne hapla*) infestations, compared with WT (Park et al., 2013; Gleason et al., 2016; Dos Santos and Park, 2019), together concurring with the conclusion that OPDA is an autonomous metabolic messenger, connecting stress cues to the readjustment of cellular redox homeostasis in actuating retro-directional signaling from the chloroplasts to the nucleus for regulating defense gene expressions.

## Cyp20-3 Relays OPDA Signaling Between Plant Defense and Growth Regulatory Pathways

Emerging evidence from a number of recent studies has underpinned that CYP20-3 is a versatile metabolite in plants, proposed to be a key regulator in controlling the interface between OPDA (defense) and light-dependent redox (growth) signaling (Dos Santos and Park, 2019, Figure 3). The latter, also known as the *e*^−^ transport chain (ETC) photosystem I (PSI), is a primary metabolism converting solar energy into biologically useful chemical energies, necessary for the production of overall biomass of plants and living organisms (Chitnis, 2001; Jensen et al., 2007). When the PSI captures solar energy, it excites *e*^−^ that reduce thioredoxins (TRXs) *via* a ferredoxin (Fd) and a Fd-TRX reductase (FTR). TRXs, small oxidoreductases, then deliver *e*^−^ and activate target enzymes in the Calvin cycle that balances consumption in photosynthesis (Meyer et al., 2009; Serrato et al., 2013; Nikkanen and Rintamäki, 2014). Recent studies, however, have revealed that TRXs can also reduce other Calvin cycle-unrelated proteins, including CYP20-3, a regulator of OPDA defense signaling (see above), and photosynthetic ETC as an *e*^−^ donor of 2-cysteine peroxiredoxin A (2CPA; Motohashi et al., 2001; Peltier et al., 2006; Laxa et al., 2007). Plastid 2CPA is a thiol-based peroxidase involved in protecting and optimizing photosynthesis. When arrived at the chloroplasts, 2CPA is activated by either different *e*^−^ donors such as NADPH-dependent TRX reductase C (NTRC), TRXs, and CYP20-3, or—as recently proposed—oxidation folding with GSH (also called S-glutathionylation), which in turn reduces toxic by-products (e.g., H_2_O_2_) in photosynthesis or activates Calvin cycle enzymes such as a fructose 1,6-bisphosphatase (Konig et al., 2002; Peltier et al., 2006; Caporaletti et al., 2007; Laxa et al., 2007; Muthuramalingam et al., 2009; Liebthal et al., 2016; Pérez-Ruiz et al., 2017; Liu et al., 2020).

In this context, OPDA binding promotes the interaction of CYP20-3 with TRXs (e.g., type-f2 and x; Cheong et al., 2017), illuminating a mode of OPDA/CYP20-3 signaling in transferring *e*^−^ from TRXs to 2CPA and/or SAT1 (Figure 3, orange arrow). The latter then stimulates plastid sulfur assimilations (e.g., GSH and thiol accumulations), which coordinate redox-resolved nucleus gene expressions in defense responses against biotic and abiotic stresses (Park et al., 2013), while accelerating the S-glutathionylation (activation) of 2CPA that promotes photosynthetic energy productions (Liu et al., 2020), postulating that OPDA/CYP20-3 signaling optimizes the growth, reproduction, and survival of plants under constant environmental stresses. Traditionally, the cost of resistance (often referred to as growth and defense tradeoff) has been typically described as a teeter-totter model where for defense to increase, growth must decrease and vice versa (Huot et al., 2014). This model well circumstantiates the response of plants to the persistent and excess surge of environmental stresses. However, in nature, plants are more often situated to encounter a consistent array of temporal and modest levels of environmental changes, while concurrently trying to ensure normal growth and developmental processes, in order to maximize their yields and production. For instance, a sorghum inbred line tolerant to sugarcane aphid (*Melanaphis sacchari*) accumulates significantly higher levels of OPDA, but little if any increase in JA/JA-Ile levels, compared to susceptible lines, as supporting insect growth (tolerance, defense) while concomitantly maintaining enhanced photosynthesis (growth, Grover et al., 2020a). Hence, recent studies have begun to elaborate an alternative model, “growth and defense coordination,” wherein a balancing act between growth and defense can synergistically optimize plant fitness (Kliebenstein, 2016). In agreement, plants' acclimations toward environmental changes and pressures causing oxidative stresses (e.g., insect and pathogen attack, tissue injury, excess light and temperature, and drought and salinity) accompany the accumulation of OPDA on a time sale of hours with attendant accumulation of reduced, non-protein thiols (Riemann et al., 2003; Kazan and Manners, 2011; Noctor et al., 2011; Savchenko and Dehesh, 2014; Hazman et al., 2015; Muench et al., 2016). This initial response subsequently reprograms cellular redox homeostasis and elevates net photosynthesis and nutrient acquisition, together providing plant tolerance to constant and multitudinous biological/ecological constraints (Koch et al., 2016).

## Signaling of OPDA in the Plant Acclimation and Adaptation of Heat Shock (HS)

In parallel, our recent study has unveiled that OPDA/CYP20-3 signaling also conveys *e*^−^ transfers toward peroxidatic (S-glutathionylated) 2CPA (2CPA^GS^) under HS stress (Figure 3, green arrow). The deglutathionylation then dimerized and inactivated 2CPA that removes HS- and PSI-induced H_2_O_2_ bursts and/or activates Calvin cycle enzymes. Hence, it enables the actuation of oxidative stress (defense) signaling and potentially counteracts plant growth, to some extent, elucidating the mode of OPDA in growth and defense tradeoff (Dave et al., 2011; Hazman et al., 2015). On the other hand, HS promoted the increased accumulation and S-glutathionylation of, in part through OPDA/CYP20-3 signaling, 2CPB (another 2CP isoform in the chloroplasts) that constitutes a stable, decameric conformation conferring a chaperone activity (Liu et al., 2020). The expression and activation of a battery of molecular chaperones (i.e., heat shock proteins, HSPs) in protecting native folding and/or assembly of cellular proteins are major defense machinery in plant acclimations of HS (Finka et al., 2011), supporting the critical role of OPDA signaling in thermotolerance (Dave et al., 2011; Muench et al., 2016; Monte et al., 2020). In Arabidopsis when HS occurred, OPDA is mainly, but not JA and JA-Ile, accumulated in the leaves and is only able to dramatically induce HSPs (Taki et al., 2005; Mueller et al., 2008; Balfagón et al., 2019). These HSPs are also induced in *coi1* mutants further delineating that OPDA is involved in COI1-independent HS-responsive pathways (Monte et al., 2020). As known from the previous study (Mueller et al., 2008), ORG expressions are largely dependent on TGA TFs (a group of bZIP TFs containing the TGACG motif) TFs. However, the induction of HSPs does not employ TGA TFs but rather a key HS TFs (HSFA1s) or CYP20-3 (Muench et al., 2016). CYP20-3 relays HS-responsive OPDA signaling in the regulation of cellular redox homeostasis that induces and/or stabilizes HSPs (e.g., decameric 2CPB^GS^), while deglutathionylating 2CPA^GS^ (suppressing peroxide detoxification) allowing to trigger the rapid ROS signaling, together enhancing heat tolerance in plants (Park et al., 2013; Hazman et al., 2015; Liu et al., 2020). This HS response subsequently leads to induce the short-term acquired tolerance and/or cross-defense responses against following abiotic and biotic stresses such as extreme temperatures, high light, salinity, drought, heavy metal, and insect (*Mayetiola destuctor*) herbivory (Cheng et al., 2018; Hossain et al., 2018; Balfagón et al., 2019), which highlights the vital activity of OPDA signaling in broad-spectrum, induced systemic tolerance/resistance against a wide range of environmental stresses, improving and optimizing growth and yield potential across economically important crops.

## Summary: Mode of Action of OPDA Signaling

As sessile organisms, plants cope with constant encounters with a wide range of biotic competitors and consumers, and abiotic constraints, through mobilizing a number of primary and secondary metabolites, and intricate signaling networks that interconnect and orchestrate large-scale changes in transcriptome, proteome, and metabolome. As described in this review, the emerging evidence has espied that OPDA is a versatile signal molecule involved in a variety of metabolic pathways, coordinating plant growth and survival in optimal condition as well as under various forms of environmental stresses (Table 1). In the recent decade, a large number of efforts have been devoted and begun to delineate the mechanistical modus operandi of OPDA signaling; thus far, three working models have been proposed. Once it is produced in the chloroplasts, OPDA is *i*) conjugated with galactolipids, GSH, and/or amino acids (e.g., Ile) before/after being released to the cytosol, in turn targets yet unknown effector/receptor proteins, and conveys ORG expressions (Böttcher and Pollmann, 2009; Ohkama-Ohtsu et al., 2011; Floková et al., 2016). Alternatively, OPDA itself can *ii*) serve as a reactive electrophile that targets and modifies thiol residues of, e.g., cysteine, histidine, and lysine in proteins (Mueller and Berger, 2009; Monte et al., 2020) triggering downstream signal transductions and metabolic cascades, or *iii*) covalently bind a CYP20-3 receptor and builds up a reduction capacity that modulates the cellular activity of oxidoreductase cascades in controlling retrograde signaling, rapidly adjusting nuclear gene expressions (Tada et al., 2008; Park et al., 2013; Cheong et al., 2017). It is, however, still unclear how these signaling mechanisms ultimately stimulate global, spatiotemporal gene expression dynamics with both distinctive and redundant transcriptional outputs. Our study suggests though that OPDA can target and fine-tune an interface between photosynthesis-derived ETC and sulfur assimilation processes in the chloroplasts (Cheong et al., 2017; Liu et al., 2020). This interplay enables plants to make an adaptive decision in allocating resources (*e*^−^) between growth and defense responses (e.g., fitness trade-offs or balances) toward different ecological challenges such as pathogens, pests, tissue injury as well as light and oxidative stresses, in the end, ensuring optimal growth, reproduction, and survival of plants. Therefore, furthering our understanding of functional and biological activities of OPDA and associated molecular mechanisms (a) will not only provide new insights into a “broad-spectrum” defense responses and (b) can enrich plant breeding and engineering strategies for the selection of elite genetic traits that will maximize plant fitness, but also (c) will address fundamental gaps in the immune activation of a mammalian system, and (d) help in improving drug developments through facilitating the rational design of more potent and safe reagents.

**Table 1:** Biophysiological activities and functions of OPDA across diverse plant genres.

**Crops**	**Defense responses**	**Growth**	**References**
*Arabidopsis thaliana*	Local defense against infections of fungal pathogens (*A. brassicacola* and *S. sclerotiarum*), insect (*B. impatiens*), and root-knot nematode (*M. hapla*). Enhanced resolution of tissue injury and tolerance to high light and heat stress.	Regulation of seed dormancy and germination Inhibition of primary root growth	Stintzi et al., 2001; Buseman et al., 2006; Mueller et al., 2008; Park et al., 2013; Dave et al., 2016; Gleason et al., 2016; Balfagón et al., 2019; Liu et al., 2020.
*Marchantia polymorpha*	Enhanced protection against heat stress.		Monte et al., 2020.
*Oryza sativa*	Local defense against insect (*N. lugens* and *M. persicae*) infections and increased tolerance toward salt stress.		Guo et al., 2014; Hazman et al., 2015.
*Populus trichocarpa*	Local defense against spider mite (*T. urticae*) infestations and enhanced adaptation of tissue injury.		Zhao et al., 2020.
*Sorghum bicolar*	Enhance tolerance to aphids (*M. sacchari*).		Grover et al., 2020a.
*Solanum lycopersicum*	Local defense against fungal (*B. cinerea*) and insect (*M. sexta* larvae) infections.	Regulation of embryo development and seed dormancy	Goetz et al., 2012; Bosch et al., 2014a; Scalschi et al., 2015.
*Solanum melongena*	Hexanoci acid-mediated systemic defense against insect (*L. decemlineata*) infestations.		López-Galiano et al., 2017.
*Triticum aestivum*	Enhanced resistance to Hessian fly (Diptera: *Cecidomyiidae*) under heat stress		Cheng et al., 2018.
*Zea mays*	Local defense against aphids (*R. maidis*) and *T. virens*-primed IST against parasites (*C. graminicola*).		Varsani et al., 2019; Wang et al., 2020.

## Author Contributions

SWP and WL, designed and wrote the article. All authors contributed to the article and approved the submitted version.

## Conflict of Interest

The authors declare that the research was conducted in the absence of any commercial or financial relationships that could be construed as a potential conflict of interest.

## Publisher's Note

All claims expressed in this article are solely those of the authors and do not necessarily represent those of their affiliated organizations, or those of the publisher, the editors and the reviewers. Any product that may be evaluated in this article, or claim that may be made by its manufacturer, is not guaranteed or endorsed by the publisher.
